# Editorial: Current Trends in Exploiting Molecular Signaling in Bacteria-Host Crosstalk

**DOI:** 10.3389/fmicb.2022.911558

**Published:** 2022-05-31

**Authors:** Carmen Mariana Chifiriuc, Monica Cartelle Gestal, Alina Maria Holban

**Affiliations:** ^1^Department of Microbiology and Immunology, Faculty of Biology, University of Bucharest, Bucharest, Romania; ^2^Earth, Environmental and Life Sciences Section, Research Institute of the University of Bucharest, Bucharest, Romania; ^3^Louisiana State University Health Sciences Center-Shreveport, Shreveport, LA, United States

**Keywords:** bacterial infections, microbiota, crosstalk, biomarkers, anti-infective

Despite antibiotics and immunization discovery, as well as tremendous progress made in medicine and environmental quality control, the global burden of infectious diseases still raises important new challenges for prevention, monitoring, diagnostic, and therapy. In case of bacterial infections, we are facing an unprecedented emergence and spread of antimicrobial resistance (AMR) globally, with medical and socio- economic disastrous consequences. Moreover, the coronavirus 2019 (COVID-19) pandemic has drastically changed the healthcare practices in terms of antibiotics and disinfectants use, eventually influencing, in a still unknown way, the dynamics of AMR. Many scientists are predicting that climate change will also influence the landscape of bacterial infections in many ways, such as the global warming will facilitate the more rapid growth and thus, the survival and spread of antibiotic-resistant bacteria and the exchange of resistance genes. This global context reiterates that multi-disciplinary and internationally concerted research is urgently needed to develop novel and efficient preventive and therapeutic strategies, for continuingly improving our preparedness and response to present and future threats from bacterial infections and AMR. Bacteria are able of intercellular communication and further, to sense and rapidly adapt to their environment through coordinated, multi-cellular responses. If their environment is the human body, pathogens' ability to sense, respond and manipulate host immune responses contributes greatly to their success to concur the host. The goal of this special issue was to present the current progress and perspectives regarding pathogen-host crosstalk, in the hope to provide a fruitful platform for feeding the pipeline for the future development of vaccines and anti-infective therapies.

By successfully fulfilling the declared goal of this Research Topic, the published papers present new, important and inspiring data concerning the effectors and mechanisms involved in the bacterial pathogens-host crosstalk during the infectious process as well as in the human microbiota dialogue with the human host organism. The presented results were obtained using state of the art methodology and infection models that could serve as inspiration for further research and offer new leads for the development of novel biomarkers and therapeutic strategies. Some valuable reviews on the above-mentioned subjects are adding to the original papers. According to their subject, the papers could be divided in three categories.

The first category presents novel effectors and pathogenic mechanisms involved in the interplay between different Gram-negative and Gram-positive bacteria and their animal or human host, during the infectious process and highlight their potential for the development of novel anti-infectious strategies. Liu et al. updated the major molecules involved in cyclic guanosine monophosphate (GMP)-adenosine monophosphate (AMP) (cGAMP) synthase (cGAS), along with the adaptor stimulator of interferon genes (STING) and the effects of the cGAS-STING pathway in various bacterial infections and bacterial immunity, which may pave the way for the development of new antibacterial drugs that specifically kill bacteria without harmful effects on the host. Luo et al. reviews the mechanisms by which *Legionella pneumophila* effectors interfere with host cells ubiquitination during the infectious process. Fu et al. focused on EspF, one of the virulence factors of Enterohemorrhagic *Escherichia coli* (EHEC) O157: H7, proving that it may mediate DNA damage, by regulating the subcellular localization and phosphorylation of SMC1. Using the *Tenebrio molitor* model, Kojour et al. unveil the role of the dimeric cytokine ligand Spätzle (Spz)5 in the innate defense against *E. coli* infection. Using a transcriptomic approach, Zhang et al. shed new insights in the mechanisms and effectors of *Salmonella enteritidis* infection in ducks, that could feed the pipeline for designing new therapeutic strategies. Chu et al. demonstrated that *Salmonella Infantis* inhibited the apoptosis of infected Caco-2 cells by intermittently phosphorylating Akt, allowing sufficient time for replication, thereby causing more severe inflammation. Liu et al. raises caution on using glucocorticoids in chronic lung disease, showing that they increase the expression of syndecan-1 (SDC1) and consequently, *Pseudomonas aeruginosa* binding to the airway epithelium. Ding et al. have shown that *P. aeruginosa* infection increases IL-17 production, augmenting inflammation in chronic obstructive pulmonary disease (COPD) patients and COPD mouse models and recommend IL-17A as a potential therapeutic strategy in controlling the outcomes of *P. aeruginosa* infection in COPD patients. Tang et al. reported for the first time a new mechanism by which *Aeromonas hydrophila* evades from host antibacterial defense by intervening CD80/86 signal, that could be targeted for the development of new therapeutic interventions. Gao et al. investigated the role of flagellin B from *Vibrio anguillarum*, an opportunistic pathogen of aquatic animals, on cell apoptosis, TLR5 expression, and production of IL-8 and TNF-α, in the perspective of future design of flagellin-based vaccines. Tan et al. offers the readers a fresh perspective regarding the correlation between the agr polymorphism and *S. aureus* pathogenic potential, while Smyth and Sun propose that Protein Kinase R (PKR), already well known as an important target in cancer, metabolic disorders, neurodegeneration, and antiviral defense, could be also be considered a promising lead for novel treatment strategies in bacterial infections. Park et al. have shown that most oral streptococci induce ROS production and subsequent apoptosis in human periodontal ligament cells, thus contributing to the progression of inflammatory conditions. Ermel et al. show that dysgeusia in COVID-19 is related to the salivary levels of innate immune response molecules such as the toll-like receptor-4, peptidoglycan recognition protein, and sACE2 and perturbation of oral biofilm.

The second batch of papers are moving the readers' focus to the human microbiota- host organisms crosstalk in health and disease. Ding et al. reviewed the role of microbiome and gut–brain axis in epilepsy, highlighting the possible pathogenic mechanisms and the novel therapies targeting the gut microbiota, Yuan et al. summarized the role of gut microbiota in acute central nervous system injuries, while Zheng and Wang offer the reader an update of molecular mechanisms by which gut microbiota-derived signals modulate liver injury and regeneration and the development of gut microbiota-based therapies. Zhang et al. have shown that the major cytotoxic T-cell trafficking chemokines (CTTCs) and chemokine-associated microbiota profiles are different in colorectal cancer (CRC) tumor vs. adjacent normal tissues, Yang et al. provide a detailed analysis of the oral microbiome in patients with oral squamous cell carcinoma and Prucsi et al. reviewed the involvement of oral microbiota in periodontal inflammation mediated by neutrophils. Using an *in vivo* mice model, Zheng et al.' study fills the knowledge gap regarding the dynamics and roles of respiratory microbiota in the different stages of asthma and their association with chronic asthma progression. By using a multi-omics approach, Gong et al. identified intestinal microbiota, fecal and serum metabolites and cytokines profiles which could predict for anti-N-methyl-D-aspartate receptor (NMDAR) encephalitis clinical severity and relapse. All these papers could inspire other researchers for developing novel microbiota-derived biomarkers used for risk prediction, early diagnosis and monitoring of cancer and other chronic diseases.

The third category of papers refer to novel antibacterial strategies based on natural compounds and on probiotics, updating or demonstrating their intimate mechanisms of actions. He et al. proposes that melatonin could be an effective approach against various pathogenic bacterial infections, acting by decreasing formation of reactive oxygen and nitrogen species, promoting detoxification and protecting mitochondrial damage, while Yang et al., demonstrate the anti-QS activities of the phenolic compound paeonol in a *Caenorhabditis elegans* infection model. Probiotics are used to alleviate the deleterious effects of inflammatory reactions, but the intimate mechanisms of action are still to be unveiled. Using *in vitro* and *in vivo* mastitis models, Zheng et al. have demonstrated that *Lactobacillus casei*, besides its direct competition with *E. coli*, could reduce the inflammation associated with *E. coli*-induced mastitis by consolidating the tight junctions and decreasing the expression of the inflammatory cytokines. In the same infection model, Li et al. have shown that *L. rhamnosus* GR-1 prevents *E. coli*-induced apoptosis induced PINK1/Parkin-mediated mitophagy that eliminated damaged mitochondria and reduced ROS production and NLRP3 inflammasome activation. The efficiency of *Lacticaseibacillus casei* T21 was demonstrated *in vitro*, on colonic epithelial cells and *in vivo*, on a murine *Clostridioides difficile* infection model, by assessing many reliable parameters. This could represent a model study for demonstrating the therapeutic efficiency of probiotic formulations and unveil their possible mechanisms of action (Panpetch et al.). Liang et al. show that fecal microbiota transplantation (FMT) controls progression of experimental autoimmune hepatitis in mice by modulating the follicular regulatory T cells and restoring the gut dysbiosis. Moving to huma studies, Ren et al. demonstrated the long-term efficacy and safety of single-donor, low-intensity FMT in treating ulcerative colitis (UC). The patients with UC sustained remission had a higher abundance of *Bifidobacterium breve*, while a higher level of *Bacteroides* spp. was observed in the relapse group.

We are very grateful to all authors and reviewers for their valuable contributions and hope that the readers will find our Research Topic selection interesting and inspiring for their future work.

## Author Contributions

All authors have contributed to the Editorial and approved the final paper.

## Funding

This project was financed by the Ministry of Research, Innovation and Digitalization through Program 1—Development of the national R&D system, Subprogram 1.2—Institutional performance—Financing projects for excellence in RDI, project C1.2.PFE-CDI.2021-587, Contract no. 41 PFE/30.12.2021.

## Conflict of Interest

The authors declare that the research was conducted in the absence of any commercial or financial relationships that could be construed as a potential conflict of interest.

## Publisher's Note

All claims expressed in this article are solely those of the authors and do not necessarily represent those of their affiliated organizations, or those of the publisher, the editors and the reviewers. Any product that may be evaluated in this article, or claim that may be made by its manufacturer, is not guaranteed or endorsed by the publisher.

